# Sesquiterpenes from Brown Algae

**DOI:** 10.3390/md23050210

**Published:** 2025-05-15

**Authors:** Irene Moreno-Gutiérrez, Sonia Berenguel-Gómez, Manuel Muñoz-Dorado, Míriam Álvarez-Corral, Ignacio Rodríguez-García

**Affiliations:** Organic Chemistry, CeiA3, CIAIMBITAL, University of Almeria, 04120 Almería, Spain; img823@ual.es (I.M.-G.); sbg479@ual.es (S.B.-G.); mdorado@ual.es (M.M.-D.)

**Keywords:** brown algae, brown seaweed, *Phaeophyceae*, sesquiterpene, sesquiterpenoids, volatile organic compounds

## Abstract

Algae are the group that has managed to generate the largest number of compounds and secondary metabolites with different properties, many of them only present in the aquatic kingdom. Among them, brown algae are one of the main producers within marine ecosystems. Furthermore, one of the main groups of secondary metabolites studied are sesquiterpenes due to the great variety of properties observed, largely due to the great structural variability of these compounds. Many studies have been carried out to isolate and characterize compounds with a sesquiterpene structure from different species of brown algae. This article reviews the natural occurrence of sesquiterpene and derivatives in brown algae. A total of 51 sesquiterpenes isolated from brown algae, having monocyclic, bicyclic, or tricyclic skeletons, as well as 23 sesquiterpenoids with various chemical structures, are depicted. Moreover, there are at least eleven publications focused on the study of the profiles on volatile organic compounds (VOCs) within brown algae, derived using several analytic and extraction techniques, and in the finding of a large variety of structures of sesquiterpenes.

## 1. Introduction

Marine organisms constitute one of the most important sources of secondary metabolites with bioactive properties [1]. These types of marine natural products have been found in algae, sponges, mollusks, cnidarians, bryozoans, and other organisms [2].

Algae constitute up to 10% of the plant kingdom and account for 40% of global photosynthesis [3]. They are mainly present in seas and oceans, although they can also be found in lakes, freshwater ponds, soil, and rocks. This group of marine organisms has provided the greatest number of new secondary metabolites [4]. Natural products research on algae began in the 1970s, with studies highlighting metabolites, toxins, ecology, and biodiesel [5], often discovering complex compounds not found in the terrestrial world [3]. Although studies have primarily focused on the application of algae as a form of bioenergy, recent research into new uses of algae in industrial sectors such as pharmaceuticals and health has increased exponentially, accompanied by advances in characterization techniques, analysis, and bioactivity detection. The richness and chemical diversity of algae are due to the taxonomic and genetic differences between species and groups of algae, as well as the geographical distribution of the specimens [6].

Macroalgae are classified into three large classes: green algae (Chlorophyta), brown algae (Phaeophyta), and red algae (Rhodophyta) [7]. Among them, brown algae stand out as one of the most important production groups in coastal marine ecosystems [8]. There are about 2000 known species of brown algae, grouped into 270 genera and 13–19 families [9].

Brown algae can be classified into different families: *Laminaria*, *Dictyota*, *Sargassum*, *Ectocarpus*, and *Fucus* [10]. The traditional classification of brown algae, throughout the 20th century, was based on a combination of life cycle structure, thallus architecture, and gametic traits. However, molecular phylogenies changed their phylogenetic structure [11]. Recently, the study of secondary metabolites isolated from organisms as taxonomic markers has proven to be very useful in the phytochemical, phylogenetic, and ecological studies of algae [12], always considering that there are several environmental factors that can affect the production of these compounds [13].

One of the most studied types of secondary metabolites, due to their structural variety and the biological properties observed in certain examples, are sesquiterpenes. Within the marine system, sesquiterpenes have mostly been isolated from sponges, although there are a multitude of examples isolated from brown algae, as well as other organisms [14].

## 2. Sesquiterpenes and Sesquiterpenoids in Brown Algae

In this paper, sesquiterpenes have been classified according to the complexity of their structure, starting with the number of rings, as monocyclic, bicyclic, and tricyclic, since no examples of linear or acyclic sesquiterpenes have been found in this class of macroalgae. In addition, we have included a fourth group named brown algae sesquiterpenoids. Under this category, we have included those compounds in which the sesquiterpene skeleton appears linked to an aromatic fragment (usually a phenol or a benzoic acid) or a hydroquinone. We have also included here those with a structure of bisnorsesquiterpenes, C13 compounds resulting from the loss of two carbon atoms from the standard C15 sesquiterpene skeleton. Within each of the main groups, the classification has continued tending to the main structural characteristics of each class of molecules, including type of skeleton, position of functionalization, and/or functional groups present in the structure. All these compounds are covered in Section 2.1, Section 2.2, Section 2.3 and Section 2.4. These structures have been determined by combined spectroscopic techniques, with nuclear magnetic resonance imaging being the most used. Some were described as new natural products at the time of isolation from brown algae.

There are also several studies discussing the identification of volatile compounds using mass spectrometry combined with gas chromatography. Section 2.5 is devoted to the description of these compounds. However, as the structures of the compounds in this section were not elucidated in the studies of brown algae, our attention is focused on the extraction techniques used, which in this case play a crucial role.

### 2.1. Sesquiterpenes and Sesquiterpenoids with Monocyclic Skeleton

Seven sesquiterpenes or sesquiterpenoids containing one ring in their structure have been found in brown algae. Six of them are part of the germacrane family, while the other one has a bisabolol structure.

- Germacranes

**Figure 1:**
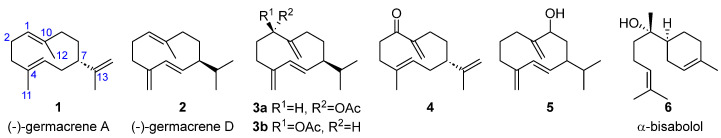
Monocyclic sesquiterpenes and sesquiterpenoids from brown algae with germacrane skeleton (**1**–**5**) and bisabolane skeleton (**6**).

Among the six compounds with the characteristic ten membered monocyclic germacrane skeleton that have been isolated from brown algae, only two non-oxygenated substances with a germacrene structure have been described. In 1990, Segawa et al. isolated, from the methanol extracts of the fresh algae *Dyctiopteris divaricata*, the already known compound (−)-germacrene A (**1**) (Figure 1) [15], and in 1994, De Rosa et al. isolated (−)-germacrene D (**2**) from the brown alga *Taonia atomaria* from the North Adriatic sea [16].

The other germacrane derivatives that have been found in brown algae have oxygenated functions in C1 or C9 (Figure 1). In this way, two epimeric C1-acetylated germacrane structures have been found in two different algae: (1*S*,7*R*)-1-acetoxygermacra-4(11),5(6),10-triene (**3a**), a possible biosynthetic precursor of the sesquiterpenes with cadalane skeleton, was isolated from *Dilophus fasciola* [17], while its epimer (1*R*,7*R*)-1-acetoxygermacra-4(11),5(6), 10-triene (**3b**) was obtained from the brown algae *Taonia atomaria* [18]. In addition, one substance with a germacrane structure incorporating a carbonyl group in C1, germacra-5,10,13-trien-1-one (**4**), was isolated from *Dictyopteris divaricata* [15]. Only one C9-oxigenated germacrane structure has been isolated from brown algae, germacra-4(11),5,10(12)-trien-9-ol (**5**), which was described by Othmani et al., in 2016, when studying the extracts from *Taonia atomaria* [18].

- Bisabolol

A bioassay-guided study of the methanol extract of *Padina gymnospora* performed in 2015 proved the presence of *α*-bisabolol (**6**) (Figure 1) as an active lead, with a remarkable relative abundance of 69% [19]. The study proved that both the methanol extract and the isolated sesquiterpenoid **6** mitigate biofilm formation and quorum sensing controlled virulence factor production of the nosocomial pathogen *Serratia marcescens*. Three years later, the same research group studied the neuroprotective effect of the acetone extract of *P. gymnospora* as well as pure α-bisabolol as a part of their studies on the inhibition of amyloid beta (A*β*) peptide development and aggregation during an Alzheimer’s disease study, which concluded that both the acetone extract and pure bisabolol have neuroprotective effect against A*β* mediated Alzheimer’s disease pathology [20].

Table 1 summarizes the distribution of monocyclic sesquiterpenes and sesquiterpenoids in brown algae.

### 2.2. Sesquiterpenes and Sesquiterpenoids with Bicyclic Skeleton

Bicyclic sesquiterpenes and sesquiterpenoids are the most abundant in brown algae, as 39 sesquiterpenes containing 2 carbocycles in their structure have been described: 7 with selinene core skeleton, 25 with cadinene skeleton, 1 with a spiroaxane structure, and 6 oplopane derivatives.

- Selinanes

**Figure 2:**
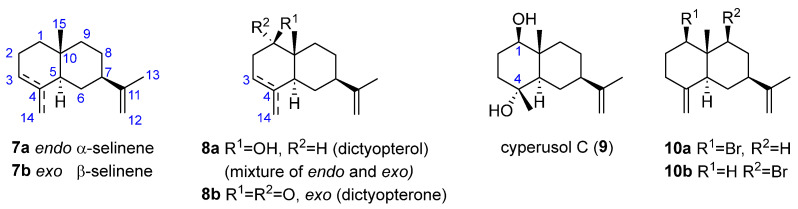
Bicyclic sesquiterpenes and sesquiterpenoids from brown algae with selinane skeleton.

The selinane skeleton has a *trans*-decalin moiety (Figure 2). Among the seven selinane sesquiterpenes isolated from brown algae, two are non-oxygenated compounds, *α*-selinene (**7a**) and *β*-selinene (**7b**), which differ in the position of one double bond. Both were isolated from *Dictyopteris divaricata* in 2009 by Ji et al. [21]. Different functionalized derivatives, mainly oxygenated and halogenated, have also been found. In this regard, two C1-oxygenated structures were isolated from the essential oil of *Dictyopteris divaricata* Okamura (“Yezoyahazu”) in 1966 by Kurosawa et al. as an inseparable mixture of isomers differing in the position of a double bond: C3-C4 (*endo*) and C4-C14 double bond (*exo*). They named the mixture dictyopterol (**8a**, Figure 2) [22]. In addition, the ketone selinene-1-one (also named dictyopterone, **8b**) was also present in the essential oil. In a different study, the C1,C4 di-oxygenated selinene, 1,4-dihydroxyselin-11-ene, named cyperusol C (**9**), was obtained from *Dictyopteris divaricata* [21]. Furthermore, two different halogenated derivatives differing in the position of the bromine substituent, 1-bromoselin-4(14),11-diene (**10a**) and 9-bromoselin-4(14),11-diene (**10b**), were also isolated and identified from *D. divaricata* by the same authors [21].

- Cadinanes

**Figure 3:**
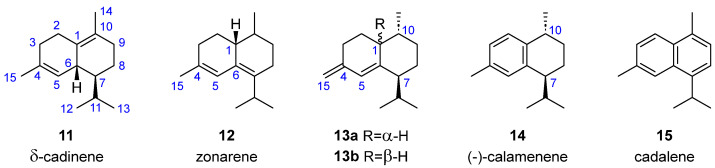
Non-oxygenated cadinenes from brown algae.

Among the 25 substances with a cadinane structure isolated from brown algae, we can distinguish a group of molecules without oxygen functions, comprising 6 molecules. *δ*-Cadinene (**11**, Figure 3), with a basic cadinane structure featuring two non-conjugated double bonds, was isolated from *Dilophus fasciola* in 1979 by Amico et al. [23], who studied the less polar fractions of the chloroform extract of the algae. In 1995, Tringali et al. re-isolated this compound while re-investigating a species previously misidentified as *Dilophus fasciola*, now recognized as *Taonia atomaria* f. *ciliata* [24].

Three cadinane structures with conjugated double bonds have been obtained from brown algae. Zonarene (**12**) was the first example of a conjugated diene among the cadinane hydrocarbons and was identified as the main hydrocarbon component of *Dictyopteris zonaroides* by Fenical et al. in 1972 [25]. Two epimeric 4-cadinene compounds were isolated from different algae: (1*R*)-cadinane-4(15),5-diene (**13a**) was isolated in 1979 by Amico et al. from *Dilophus fasciola* [23], and its epimer, (1*S*)-cadinane-4(15),5-diene (**13b**), was isolated in 1994 by De Rosa et al. from a sample of *Taonia atomaria* collected in the North Adriatic Sea [16].

Furthermore, two structures containing aromatic rings have also been found in different algae. (10*R*)-*trans*-(−)-calamenene (**14**), which has one aromatic ring in its skeleton, was identified in *Dilophus fasciola* in 1979 when Amico et al. studied its chloroform extract [23]. This compound was also found in *Taonia atomaria* when Othmani et al. analyzed this seaweed extract in 2016 [18]. Cadalene (**15**), which contains two conjugated aromatic rings, was isolated from *Dictyopteris divaricata* in 2009 by Ji et al. [21].

Nineteen sesquiterpenoids with the cadinane skeleton, featuring various oxygenated functional groups, have been isolated from brown algae. The most common oxygenation positions are C1, C3, C5, C10, and C11. Based on the degree of oxygenation, the structures can be classified into five groups: mono-, di-, and trioxygenated compounds, *α*,*β*-unsaturated carbonyl compounds, and molecules containing an epoxy or peroxy ring.

**Figure 4:**
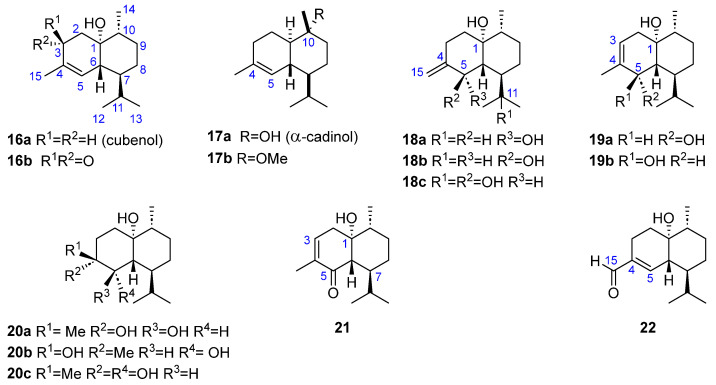
Oxygenated cadinenes from brown algae.

Three monooxygenated cadinanes have been identified. The first one, cubenol (**16a**, Figure 4), which has a hydroxyl group at C1, has been found in *Dilophus fasciola* [23], *Taonia atomaria* f. *ciliata* [24], *Dictyopteris delicatula* Lamaouroux [26], and *Dictyopteris divaricata* [27]. Two other molecules have an oxygenated function at the C10 position: α-cadinol, also named (±)-torreyol (**17a**), which was isolated from *Dictyopteris delicatula* Lamaouroux [26] and from *Dictyopteris divaricata* [27], while *α*-cadinol methyl ether (**17b**) was identified in the extract of *Taonia atomaria* [18].

Four dihydroxylated structures have been described, all of them with the functional groups in positions C1 and C5 and differing only in the location of the double bond. With an exocyclic C4-C15 double bond, two structures can be distinguished: (+)-(1*R*,5*S*,6*R*,7*S*,10*R*)-cadinan-4(15)-ene-1,5-diol, also named cadinan-4(15)-ene-1*β*,5*β*-diol (**18a**), and (+)-(1*R*,5*R*,6*R*,7*S*,10*R*)-cadinan-4(15)-ene-1,5-diol, also named cadinan-4(15)-ene-1*β*,5*α*-diol (**18b**), both of them isolated from *Dictyopteris divaricata* [28,29]. Two additional structures, but with a C3-C4 endocyclic double bond, have been identified from the same source: (+)-(1*R*,5*S*,6*R*,7*S*,10*R*)-cadinan-3-ene-1,5-diol, also named cadinan-3-ene-1*β*,5*β*-diol (**19a**), and (+)-(1*R*,5*R*,6*R*,7*S*,10*R*)-cadinan-3-ene-1,5-diol, also named cadinan-3-ene-1*β*,5*α*-diol (**19b**) [29] (Figure 4).

Structures containing three hydroxyl groups have also been described. These molecules maintain oxygenated groups at C1 and C5, while the third hydroxy group can be located at either C4 (three molecules) or C12 (one molecule). The systems with C4-HO are: 4β,5α-dihydroxycubenol ((1*R*,4*R*,5*R*,6*R*,7*S*,10*R*)-cadinan-1,4,5-triol) (**20a**), found in *Dictyopteris delicatula* Lamaouroux [26]; 4*α*,5*β*-dihydroxycubenol ((1*R*,4*S*,5*S*,6*R*,7*S*,10*R*)-cadinan-1,4,5-triol) (**20b**), found in *Dictyopteris divaricata* [27]; and 4*β*,5*β*-dihydroxycubenol ((−)-(1*R*,4*R*,5*S*,6*R*,7*S*,10*R*)-cadinan-1,4,5-triol) (**20c**), also isolated from *Dictyopteris divaricata* [29]. Additionally, the molecule having C1, C5, and C12 hydroxy groups (**18c**), which has been described as (+)-(1*R*,5*R*,6*R*,7*R*,10*R*)-cadinan-4(15)-ene-1,5,11-triol, was also isolated from *D. divaricata* [29] (Figure 4).

Three cadinanes containing an *α*,*β*-unsaturated carbonyl group at various positions have been isolated from *D. divaricata*, all of which share a common hydroxy group at C-1. Two of these compounds are ketones: cubenol-3-one (**16b**, Figure 4) [27] and (−)-(1*R*,6*S*,7*S*,10*R*)-1-hydroxycadinan-3-en-5-one (**21**) [29]. The third *α*,*β*-unsaturated carbonyl derivative is an aldehyde: (−)-(1*R*,6*R*,7*S*,10*R*)-15-oxocadinan-4-en-1-ol (**22**) [29].

**Figure 5:**
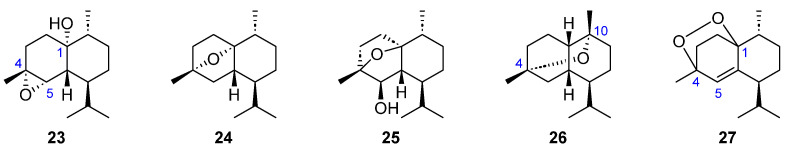
Cadinene oxides isolated from brown algae.

Finally, four additional cadinane oxides and one endoperoxide have been identified in brown algae (Figure 5). Thus, the epoxide 4*β*,5*β*-epoxycadinan-1*β*-ol (**23**) was isolated from *D. divaricata* [27]. On the other hand, the 1,4-oxide 1,4-epoxycadinane (**24**) was found in *Taonia atomaria*, while its epimer with an extra C5-hydroxy group, 1,4-epoxymuurolan-5*α*-ol (**25**), was isolated from *D. divaricata* [30]. Two additional cadinane oxides were isolated from *T. atomaria*: the 1,5-oxide 4,10-epoxymuurolane (**26**) [24] and the endoperoxide 1,4-peroxymuurol-5-ene (**27**) [18] (Figure 5).

- Spiroaxanes

**Figure 6:**
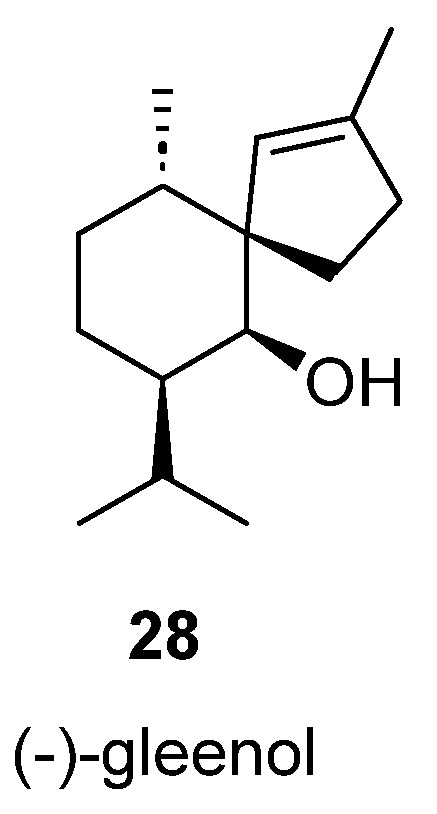
Gleenol, a spiroaxane sesquiterpenoid isolated from brown algae.

Sesquiterpenoids with a spiro [4.5]decane core are known as spiroaxanes. Only one example in this category has been described in brown algae, (−)-gleenol (**28**), which was isolated from extracts of *T. atomaria* [16,18] (Figure 6).

- Oplopanes

**Figure 7:**
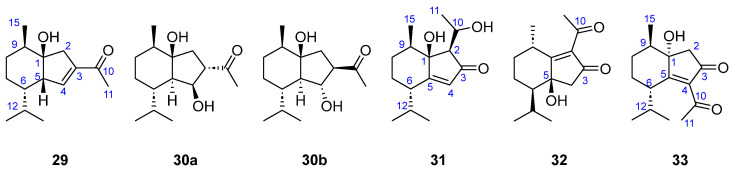
Oplopanes and analogs from brown algae.

In 2006, Song et al. described the presence of six new oplopane sesquiterpenoids (**29**–**33**, Figure 7) in the ethanolic extracts of *D. divaricata* [31]. Oplopane sesquiterpenoids usually present oxygenated functions at positions C1, C3, C4, C5, or C10. Thus, (+)-(1*R*,5*S*,6*S*,9*R*)-3-acetyl-1-hydroxy-6-isopropyl-9-methylbicyclo [4.3.0]non-3-ene (**29**) has a single hydroxy group at C1 and a carbonyl of *α*,*β*-unsaturated ketone at C10. There are three oplopane molecules with two hydroxyl groups: both diastereomeric molecules (+)-(1*R*,3*S*,4*S*,5*R*,6*S*,9*R*)-3-acetyl-1,4-dihydroxy-6-isopropyl-9-methylbicyclo [4.3.0]nonane (**30a**) and (+)-(1*R*,3*R*,4*R*,5*R*,6*S*,9*R*)-3-acetyl-1,4-dihydroxy-6-isopropyl-9-methylbicyclo-[4.3.0]nonane (**30b**) have hydroxy groups at C1 and C4 and a saturated ketone at C10, while (+)-(1*S*,2*R*,6*S*,9*R*)-1-hydroxy-2-(1-hydroxyethyl)-6-isopropyl-9-methylbicyclo [4.3.0]non-4-en-3-one (**31**) has two hydroxy groups at C1 and C10 and an *α*,*β*-unsaturated ketone at C3. The last two molecules have an *α*,*β*-unsaturated ketone and a hydroxy group. In this way, while (−)-(5*S*,6*R*,9*S*)-2-acetyl-5-hydroxy-6-isopropyl-9-methylbicyclo [4.3.0]non-1-en-3-one (**32**) has a hydroxyl group in C5 and the two C=O in C3 and C10, (−)-(1*S*,6*S*,9*R*)-4-acetyl-1-hydroxy-6-isopropyl-9-methylbicyclo [4.3.0]non-4-en-3-one (**33**) has the hydroxy group in C1 and the two C=O in C3 and C10.

Table 2 summarizes the distribution of bicyclic sesquiterpenoids in brown algae.

### 2.3. Sesquiterpenoids with Tricyclic Skeleton

**Figure 8:**
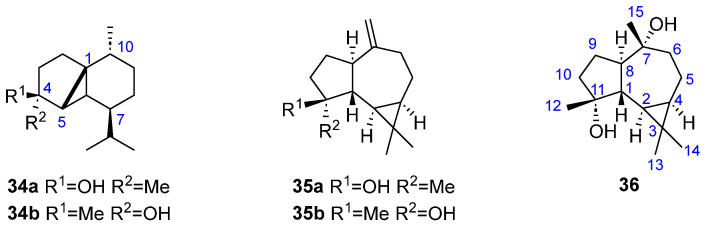
Tricyclic sesquiterpenoids from brown algae.

Five tricyclic sesquiterpenoids have been isolated and described from brown algae (Figure 8). Two of them, (−)-cubebol (**34a**) and its epimer 4-*epi*-cubebol (**34b**), were isolated in 1994 from *T. atomaria* collected from the North Adriatic Sea and both have a C-C bond between the C1 and C5 atoms of the cadinane skeleton, forming a third ring of three members, and carrying a hydroxy group in C4 position [16].

The other three molecules have a guaiane sesquiterpene skeleton that has suffered a cyclization between the isopropyl group and the adjacent carbon of the seven membered ring. Two of them are monooxigenated (Figure 8), having a hydroxy group in position C11, (+)-spathulenol [11*β*-hydroxy-11*β*,8*α*-aromadendrene (**35a**) and (+)-11-*epi*-spathulenol [11*α*-hydroxy-1*β*,8*α*-aromadendrene] (**35b**), while the other one contains an additional hydroxyl group in C7: (−)-7,11-dihydroxyaromadendrane (**36**). All of them were obtained and isolated from *Taonia lacheana* in 1995 [24]. Table 3 summarizes the distribution of tricyclic sesquiterpenoids in brown algae.

### 2.4. Other Sesquiterpenoids

- Sesquiterpene + Isoprene Unit

**Figure 9:**
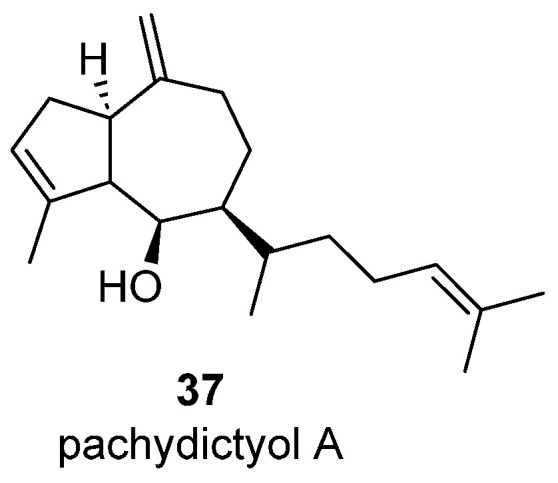
Pachydictyol A, a C15+C5 sesquiterpenoid from *Pachydictyon coriaceum*.

A new diterpene alcohol named pachydictyol A (**37**) (Figure 9) was isolated when studying the hexane extracts of the air dried *Pachydictyon coriaceum***.** The bicyclic ring system of the molecule (guaiane skeleton) is well known along sesquiterpenes but not in diterpenes, so the authors classified this substance as a sesquiterpene to which an isoprene unit has been added thorough a tail-to-tail coupling. For that reason, it is considered here as a sesquiterpenoid [32].

**Figure 10:**
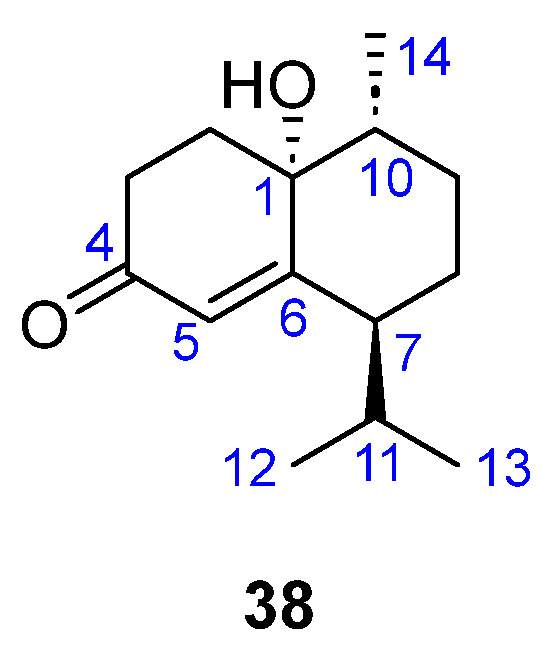
Norsesquiterpenoid (**38**) from brown *Dictyopteris divaricata*.

- Norsesquiterpenoids (C14 structure)

(−)-(1*R*,7*S*,10*R*)-1-Hydroxy-15-norcadinan-5-en-4-one (**38**) was isolated from *Dictyopteris divaricata* (Figure 10). This norsesquiterpenoid may be biogenetically derived from the oxidation of cadinane compounds as it can be seen as a dioxygenated in C1 and C5 cadinane molecule that has lost the C15 methyl (see in Figure 3 the numbering of the parent cadinene skeleton). *α*,*β*-Unsaturated carbonyl compounds are common among cadinane derivatives from brown algae [33].

- Dinorsesquiterpenoids

**Figure 11:**
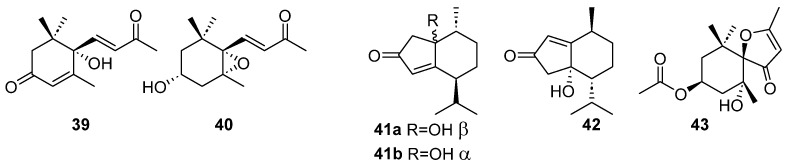
Monocyclic (**39**–**40**) and bicyclic (**41**–**43**) dinorsesquiterpenoids from brown algae.

Several dinorsesquiterpenoids (derived from the loss of two carbon units of a sesquiterpene, resulting in a C13 molecule) have been found in brown algae. We can differentiate two groups of dinorsesquiterpenes: mono- and bicyclic molecules. Among the monocyclic ones, compounds **39** and **40** (Figure 11) have been found in *Dictyopteris divaricata* [34]: dehydrovomifoliol (**39**) and 3*β*-hydroxy-5α,6*α*-epoxymegastigmen-9-one (**40**). Both compounds share an oxygenated six-membered ring substituted with three methyl groups and a side chain with an *α*,*β*-unsaturated ketone. It is reasonable to assume a common biosynthetic origin, where **40** may have been produced by intramolecular reductive nucleophilic 1,4 addition of the hydroxyl group to the *α*,*β*-unsaturated ketone of **39**, resulting in the formation of an oxirane ring.

Several bicyclic dinorsesquiterpenoids have also been found the same alga, *D. divaricata*: (+)-(1*R*,6*S*,9*R*)-1-hydroxyl-6-isopropyl-9-methylbicyclo [4.3.0]non-4-en-3-one (**41a**), (−)-(1*S*,6*S*,9*R*)-1-hydroxyl-6-isopropyl-9-methylbicyclo [4.3.0]non-4-en-3-one (**41b)**, and (+)-(5*S*,6*R*,9*S*)-5-hydroxyl-6-isopropyl-9-methylbicyclo [4.3.0]non-1-en-3-one (**42**). All three have a hydroxy group and an *α*,*β*-unsaturated ketone in the five-membered ring and might be bio-genetically derived from co-occurring compounds with cadinane skeleton by ring contraction and loss of two carbon units [34]. A new bicyclic dinorsesquiterpenoid, spheciospongone C (**43**), was also found in the extracts of *Sargassum polycystum* [35].

- Merosesquiterpenoids with aromatic rings

**Figure 12:**
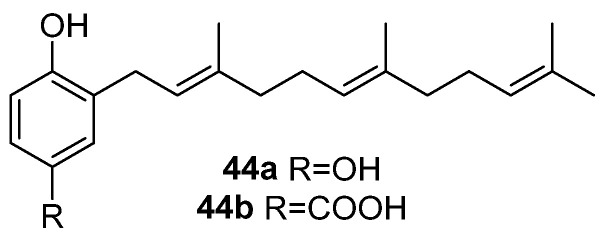
Meroterpenoids bearing a non-cyclic sesquiterpenoid fragment.

The study of methanolic extracts of *D. zonaroides (undulata)* confirmed the presence of two molecules (**44a** and **44b**) (Figure 12) characterized by a phenolic structure incorporating an acyclic farnesyl lateral chain in *ortho* to the OH group position. The first one has an aromatic ring and two hydroxy groups in -*para* relative positions: 2-(3,7,11-trimethyl-2,6,10-dodecatrienyl)-hydroquinone (**44a**). The other, 4-hydroxy-3-(1’-((2’*E*,6’*E*)-3’,7’,11’,-trimethyl-2’,6’,10-dodecatrienyl))-benzoic acid (**44b**) [36], has a COOH group instead of a OH group in the *meta* position to the side chain. Compound **44b** was first isolated from *D. divaricata* [15,33], and it was later obtained from *Padina gymnospora* in 1994 when conducting a reinvestigation of the Australian marine brown alga *Perithalia caudata* [37].

**Figure 13:**
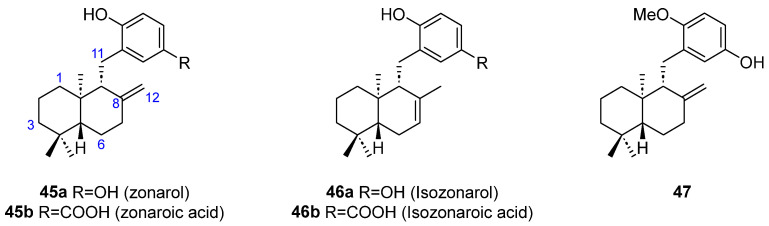
Zonarol and related hydroquinone merosesquiterpenoids from brown algae.

Thirteen cyclic sesquiterpene-substituted phenolic/hydroquinone structures (**45**–**54**) have been shown to be present in different brown algae. Five molecules having a 1,4 disubstituted phenolic group attached to a bicyclic sesquiterpene with no further oxygenations have been found in *Dictyopteris undulata* Okamura (**45**–**47**) (Figure 13). Both zonarol (**45a**) [36,38,39,40,41,42,43,44] and isozonarol (**46a**) [36,39,40,41,42] share a hydroquinone substituent linked to the C11 of an *ent*-drimane skeleton, although they differ in the position of the double bond in the sesquiterpene moiety. Zonaroic acid (**45b**) [36,38,41,43] and isozonaroic acid (**46b**) [41] are analogous structures to **45a** and **46a**, respectively, but the sesquiterpene part is attached to a 4-hydroxybenzoic acid. The monomethyl ether derivative of zonarol **47** is also present in *D. undulata* [43].

**Figure 14:**
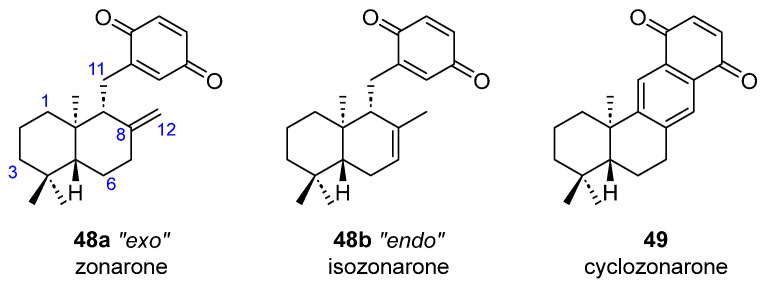
Zonarol-related quinone-merosesquiterpenoids from brown algae.

Three additional substances with a higher degree of oxidation have also been extracted from *D. undulata* (Figure 14). Now, the *ent*-drimane core has a quinone attached to the C11 position. However, while zonarone (**48a**) [40,42] has an exocyclic double bond, isozonarone (**48b**) [41,42] is the endocyclic isomer. Cyclozonarone (**49**) [42] has a tetracyclic structure possibly derived from a cyclization of zonarone.

**Figure 15:**
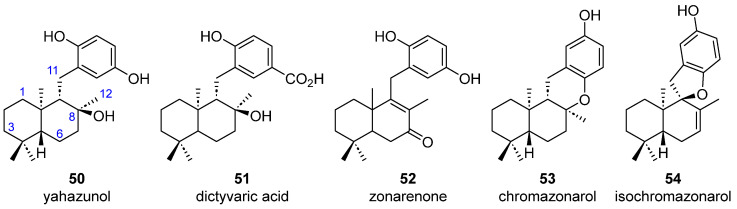
Oxygenated aromatic merosesquiterpenoids from brown algae.

The other five merosesquiterpenic structures have oxygenated functional groups on the drimane core (Figure 15). Yahazunol (**50**) [36,40,43] has been found in *D. undulata* and has a C8 hydroxy group. Dictyvaric acid (**51**) [34], found in *D. divaricata*, has a hydroxy group in the same position of the sesquiterpene core as **50** but the aromatic ring is a 4-hydroxybenzoic acid. Zonareone (**52**), found in *D. undulata* [36], has an *α*,*β*-unsaturated carbonyl in the sesquiterpenic part, which is again carrying a 4-hydroxyphenol.

In addition, there are two structures (**53** and **54**) that have a fourth ring, which is oxygenated and connects the sesquiterpene part to a phenolic group. In both molecules, the aromatic ring shows a *para*-dioxygenated pattern. While chromazonarol (**53**) has an extra six-membered ring and has been found both in *D. divaricata* [34] and in *D. undulata* [36,38,41,42,45], isochromazonarol (**54**), which has only been isolated from *D. undulata*, has a five-membered ring [36,38,45]. Absolute configurations of these meroterpenoids were reported by chemical synthesis [46,47,48] or chemical degradation [38].

Table 4 summarizes the distribution of sesquiterpenoids, nor- and dinor-sesquiterpenoids, and merosesquiterpenoids from brown algae.

**Figure 16:**
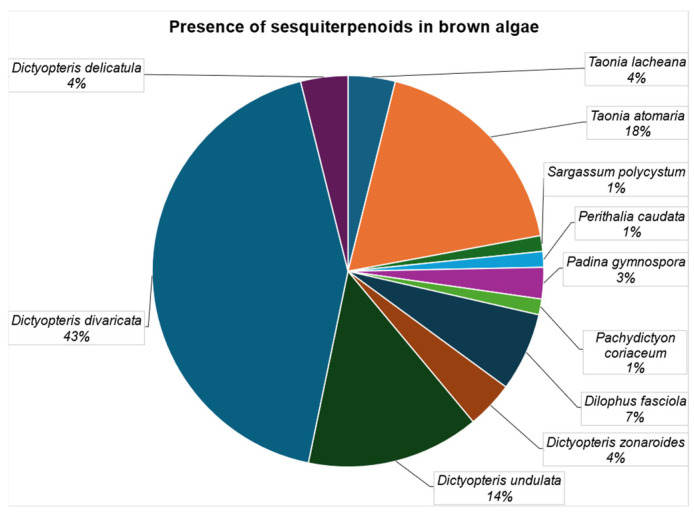
Presence of sesquiterpenoids in brown algae.

Figure 16 summarizes the presence of sesquiterpenoids in brown algae. *Dictyopteris divaricata* is the richest species, as it has provided 43% of the total number of substances isolated in brown seaweeds. The second species that has afforded more compounds is *Taonia atomaria* (18%) and the third one is *Dictiopteris undulata* (14%). These three species concentrate more than 75% of the compounds. Individually, all the others have provided less than 7% of the structures.

**Figure 17:**
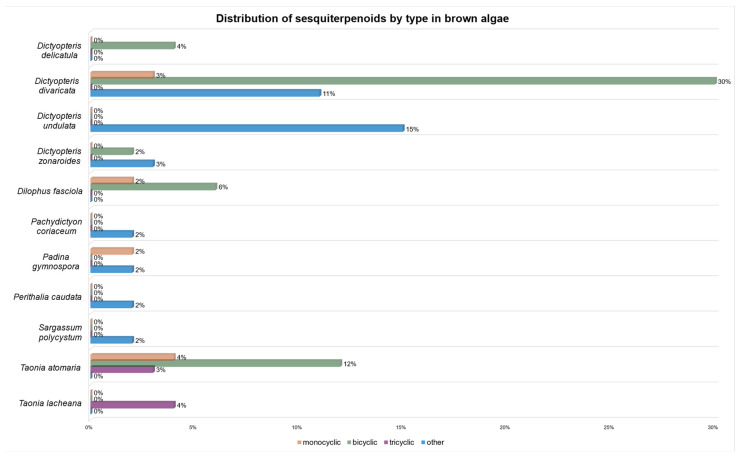
Distribution of sesquiterpenoids by type in brown algae.

Considering the category of compounds, monocyclic sesquiterpenoids are scarcely present, with *Taonia atomaria* having the highest amount (4%) (Figure 17). On the other hand, bicyclic sesquiterpenoids constitute the main group. In this case, *Dictyopteris divaricate* (30%) and *Taonia atomaria* (12%) are the richest species. Tricyclic sesquiterpenoids are also scarcely abundant. Only *Taonia lacheana* (4%) and *Taonia atomaria* (3%) have produced these kinds of substances. Finally, merosesquiterpenoids and those lacking one or two atoms, classified as “others”, are mainly present in *Dictyopteris undulata* (15%) and *Dictyopteris divaricate* (11%).

### 2.5. Volatile Organic Compounds (VOCs) in Brown Algae

Volatile organic compounds (VOCs) constitute a diverse class of secondary metabolites that play crucial roles in the ecological and physiological functions of algae. Several studies have focused on VOC analysis in brown algae (Phaeophyceae), as their VOC profiles are shown to notably differ between species and may be influenced not only by the method of extraction [49] but also by diverse seasonal factors [50,51].

Special interest has been taken in the identification and quantification of sesquiterpenes using different extraction techniques, such as hydrodistillation (HD), headspace solid-phase extraction (HS-SPME), microwave-assisted extraction (MAE), and supercritical fluid extraction (SFE) [49,52,53,54]. These techniques mainly differ in the type of compounds they can efficiently isolate and in their ability to preserve the chemical integrity of volatile metabolites.

A global view of the presence of volatile sesquiterpenes in brown algae is presented in Table 5, where the different compounds are related with their natural source through the appropriate bibliographic reference. All the detailed structures can be found in the Supplementary Materials (Figures S1–S4).

Here we present the results of VOC analyses in 13 species of brown algae from the genera *Dictyota*, *Dictyopteris*, *Halopteris*, *Cladostephus*, *Cystoseira*, *Scytosiphon*, *Taonia*, and *Padina*. The most studied species have been *Dictyota dichotoma*, with at least four recent studies between 2018 and 2023 [50,55,56,57], and *Dictyopteris divaricata* [58,60] and *D. prolifera* [57,58,60]. Regarding composition, a rather notable qualitative and quantitative variability has been observed in the sesquiterpene profiles. However, some compounds do appear recurrently, such as germacrene D (**2**), cubenol (**16a**), gleenol (**28**), epi-bicyclosesquiphellandrene (**85**), and *δ*-cadinene (**11**), with germacrene D (**2**) being one of the most abundant, especially in *D. dichotoma* [50,55,56,57] and *Taonia atomaria* (up to 62% in HS-SPME) [53]. Notable differences between techniques have also been described [49]. It is important to underscore the structural diversity of sesquiterpenes in brown algae and to accentuate the importance of carefully selecting the extraction method to obtain representative profiles.

*Dictyota dichotoma* is the most studied species, for which various studies have used the HS-SPME/GC-MS technique, showing consistent but also complementary results. Jerkovic et al. described in 2018 that sesquiterpenes are the major identified class of compounds in this species [56]: germacrene D (**2**) (28.3%), bicyclogermacrene (**72**) (4.7%), and several sesquiterpenes with cadinenyl, muurolenyl, and amorphenyl structures such as *δ*-cadinene (**11**) (8.3%), *γ*-cadinene (**93**) (3.4%), *β*-cadinene (**92**) (2.8%), *trans*-cadina-1,4-diene (**89**) (1.2%), *epi*-zonarene (**109**) (4.3%), *β*-bourbonene (**119**) (5.1%), *α*-copaene (**121**) with lower percentage, *α*-muurolene (**105**) (2.2%), *γ*-muurolene (**106**) (2.1%), and *α*-amorphene (**83**) (3.5%). Cadinenyl structures are much more abundant than muurolenyl and amorphenyl ones, because of its thermodynamically unfavorable *cis*-decalin skeleton. Later, in 2021, these results were confirmed, describing germacrene D (**2**) again as the most abundant (34.83% in DVB/CAR/PDMS fiber and 62% in PDMS fiber) along with other previously identified ones as β-cadinene (**92**), γ-cadinene (**93**), δ-cadinene (**11**), α-muurolene (**105**), γ-muurolene (**106**), and bourbonene (**119**) [57]. An even more detailed study was carried out in 2022 by Radman et al. [50], where the authors evaluated the seasonal variability in the composition of *D. dichotoma* during the months of May to September, using HS-SPME on two fibers (I and II) and hydrodistillation (HD). The VOCs content differences were found to be higher when comparing the month of May with the others. In addition, May was characterized by a larger abundance of sesquiterpenes. Several cadinene type sesquiterpenes were found in May: *epi*-cubenol (**100**) (11.8% (I); 2.6% (II)), *δ*-cadinene (**93**) (6.4% (I); 3.5% (II)), *α*-amorphene (**83**) (3.4% (I); 2.5% (II)), *epi*-bicyclosesquiphellandrene (**85**) (2.9% (I); 2.3% (II)), *γ*-cadinene (**93**), cadina-1,4-diene (**88**), *τ*-cadinol (**96**), *α*-copaene (**121**), *α*-cubenene (**123**), *β*-cubenene (**124**), *γ*-curcumene (**64**), *γ*-muurolene (**106**), *α*-calacorene (**97**), *τ*-cadinol (**96**), and cubenol (**16a**). The presence of germacrane structures was also notable: germacrene D (**2**) (6.1% (I); 14.4% (II)), germacrene C (**70**) (3.3% (I); 4.6% (II)), and selinane-type sesquiterpenes: *α*-selinene (**81**) (1.9% (I); 0.8% (II)), aromadendrane- type sesquiterpenes: *β*-gurjunene (**115**) (5.7% (I); 3.9% (II), caryophyllene-type sesquiterpenes: *α*-humulene (**71**) (5.85% (I); 3.9% (II)), and others: gleenol (**28**) (1.1% (I); 0.7% (II)). Fresh *D. dichotoma* VOCs when using HD are different, noticing gleenol (**28**) as the most abundant sesquiterpene in May (3.8%) and finding other sesquiterpenes in lower abundance and number than in HD case. On the other hand, some compounds, such as (*Z*, *E*)-farnesyl acetate (1.3%), (*Z*,*E*)-farnesol (1.4%), caryophyllene oxide (0.1%), (*E*)-*β*-guaiane (0.3%), or α-guaiol (0.3%), were only found in HD profiles.

De Grazia et al. described a preparative three-dimensional GC and nuclear magnetic resonance for the isolation and identification of sesquiterpenic VOCs from *D. dichotoma* extracted by hydrodistillation. They managed to characterize new ether-type sesquiterpenes and highlighted the complexity of the chemical profile of this species [55]. The evaluation of the composition of the hydro-distilled extract of *D. dichotoma* by GC-MS and GC-FID found sesquiterpenes as the main components in the sample (29 compounds found): *cis*-4,10-epoxy-amorphane (**127**) (36.2%), zonarene (**12**) (10.7%), *trans*-muurola-4(14),5-diene (**103**) (8.1%), *γ*-amorphene (**84**) (7.4%), or cubenol (**16a**) (5.6%). Two unknown compounds, which accounted for about 19.29% and 36.2%, respectively, were investigated and their structures were elucidated by NMR and theoretical calculations as the cadinene oxides **24** and **26** (Figure 5).

Other species of the genus *Dictyopteris* have also been studied, showing a high abundance of different sesquiterpenes. One of these species, *Dictyopteris membranacea*, was studied in 2007 by El Hattab et al. The authors used three different extraction methods (HD, MAE, and SFE) [49] and analyzed the oils by GC–MS. Sesquiterpenes were found to be the main chemical class of compounds in the MAE extracts, although they were not present in HD and SF oils). The most abundant sequiterpenes were albicanol (23.1%), zonarene (**12**) (5.6%), axenol (6.0%), *epi*-bicyclosesquiphellandrene (**85**) (6.3%), and α-cubebene (**123**) and *β*-cubebene (**124**) (2.8%). This contrasts with the large amount of sesquiterpenoids usually identified in SFE and HD oils in terrestrial plant essential oils.

On the other hand, Kajiwara et al. found the characteristic odoriferous oils from freshly wet *Dictyopteris divaricata* to be a mixture of sesquiterpenes and non-isoprenoids C11-hydrocarbons by combined GLC-MS [60]. Among them were *α*-copaene (**121**), *β*-cubebene (**124**), and *δ*-cadinene (**11**). The same authors complemented the study by describing, as major components of the essential oil of this algae, cubenol (**16a**) (over 95%), also finding other minor sesquiterpenes such as *β*-elemene (**65**) or germacrene D (**2**) [58]. Analysis by GLC-MS of the chemical composition of two other species, *D. prolifera* and *D. undulata*, revealed that the characteristic odoriferous oils from freshly wet material [58] was due to a mixture of sesquiterpenes and non-isoprenoids C11-hydrocarbons. Among them are *α*-copaene (**121**), *β*-cubebene (**123**), and *δ*-cadinene (**11**). Cubenol (**16a**) was also found in small quantities (1.5%) in *D. prolifera* [58].

In addition to the genus *Dictyopteris*, other species of the order Dictyotales have been studied for their VOCs. Thus, *Cladostephus spongiosus* was analyzed by Radman et al. [51]. The algal volatile organic compounds (VOCs) were obtained by both headspace solid-phase microextraction (HS-SPME) and hydrodistillation (HD) and analyzed by GC-MS [51], revealing the presence of sesquiterpenes (germacrene D (**2**), *epi*-bicyclosesquiphellandrene (**85**), and gleenol (**28**)), among others. The seasonal study showed that monoterpenes and sesquiterpenes exhibit the largest proportion in the May dry sample.

Both *Padina pavonica* and *Taonia atomaria* were studied by Jerkovic et al. in 2019 using HD and HS-SPME and GC-FID or MS-FID [53]. In the HS extract of *P. pavonica*, they found sesquiterpenes such as (*E*)-*β*-farnesene (**56**), *β*-bisabolene (**61**), *α*-farnesene (**55**), and *cis*- and *trans*-calamenene (**98, 99**), while in the HD oil components different sesquiterpenes, such as *trans*-*α*-bergamotene (**79**), *epi*-*β*-santalene (**78**), *α*-humulene (**71**), *β*-santalene (**77**), (*E*)-*β*-guaiene (**74**), *β*-bisabolene (**61**), and (*E*)-*α*-bisabolene (**60**), were present. The main components of the headspace and volatile oils of *Taonia atomaria* are germacrene D (**2**) (32.06% in PDMS/DVB fiber and 27.9% in DVB/CAR/PDMS fiber), the cadinane-type bicyclic sesquitepene *epi*-bicyclosesquiphellandrene (**85**) (27.5%; 25.1%), the cubebane-type tricyclic sesquiterpenes *β*-cubebene (**124**) (12.8%; 10.7%), and sesquiterpene alcohol gleenol (**28**) (9.7%; 11.1%). Minor constituents are cadina-3,5-diene (**86**) (2.45 %; 3.6 %), *trans*-cadina-1(6),4-diene (**89**) (1.2%; 2.4%), zonarene (**12**) (2.3%; 2.5%), and *α*-cubebene (**123**) (2.5%; 2.5%). On the other hand, the hydrodistillate was predominantly consistent for germacrene D (**2**) (22.2%), *epi*-bicyclosesquiphellandrene (**85**) (20.8%), and gleenol (**28**) (15.4%), with smaller percentages of other cadinane and selinane sesquiterpenes. Other sesquiterpenes were found in the distillate (not present in the headspace) such as *trans*-*α*-bergamotene (**79**), *α*-amorphene (**83**), junenol (**2**), di-*epi*-1,10-cubebol (**120**), or *δ*-cadinol (**95**).

From the order Fucales, three different algae have been studied. Bouzidi et al. studied the volatile fraction of *Cystoseira sedoides* (Cystoseiraceae family) [52], prepared through three extraction methods: HD, focused microwave assisted hydrodistillation (FMAHD), and SFE. The volatile fractions were analyzed by GC-FID-MS, finding six chemical classes of compounds: fatty acids, hydrocarbons, monoterpenes, sesquiterpenes, diterpenes, and a mixture of other chemical classes, with sesquiterpenes being the second largest group after the fatty acids, with global amounts of 8.2% by SFE, 36.7% by FMAHD, and 26.5% by HD. The main sesquiterpene found was peculiaroxide (**XX**) (11.5% FMAHD). Gleenol (**28**) is only present in SFE and FMAHD oils and axenol (**XX**) is only present in HD oil, a fact that the authors attributed to an epimerization of gleenol (**28**) because of the high temperatures in the hydrodistillation.

Sesquiterpenes have also been found among the genus *Halopteris* (Sargassaceae family). In *H. filicina*, Jerković et al. reported 26 sesquiterpenes in low percentages such as germacrene B (**2**) and *trans*-*γ*-bisabolene (**72**) when using HS-SPME (DVB/CAR/PDMS fiber (f1) and PDMS/DVB fiber (f2)) and GC-MS/FID [56]. In *H. scoparia*, they found significant differences between VOCs from fresh or air-dried algae both by HS-SPME and HD [54]. Gleenol (**28**) was detected in higher percentages in May in HD from fresh samples (6.3%), decreasing its presence in later months. Germacrene D (**2**) was only detected in HS-SPME, being mostly abundant in May (5.4%, f1; 1.9%, f2). Several sesquiterpenes were found in HS dry samples: *α*-cubebene (**123**) (0.7%), *β*-bourbonene (**119**) (0.9%), or *β*-cubebene (**124**) (1.10%), and in HS fresh samples, *δ*-cadinene (**11**) (1.1%).

From the order Ectocarpales, analysis of the essential oil from *Scytosiphon lomentaria* by GC and GC-MS revealed sesquiterpenoids as main constituents (ca 26%). Among them were *δ*-cadinene (**11**), cubenol (**16a**), *epi*-cubenol (**100**), and *β*-elemene (**65**) [59].

**Figure 18:**
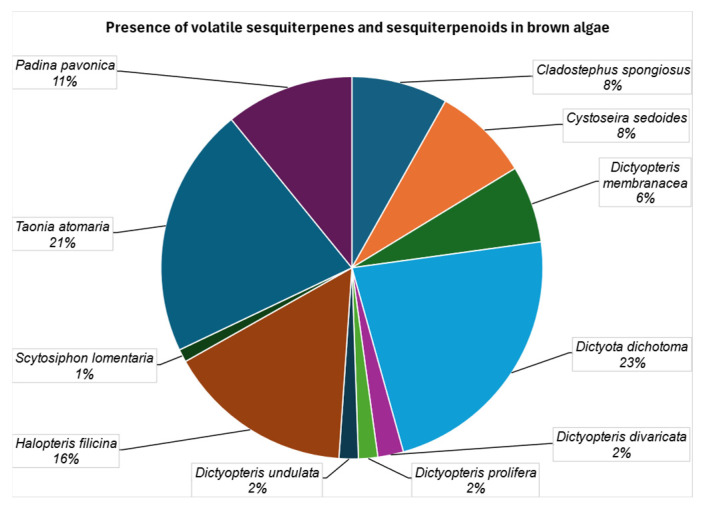
Presence of volatile sesquiterpenes and sesquiterpenoids in brown algae.

Figure 18 summarizes the presence of volatile sesquiterpenes and sesquiterpenoids in brown algae. *Dictyota dichotoma* (23%), *Taonia atomaria* (21%), and *Halopteris filicina* (16%) are the species that have provided the largest number of sesquiterpenes and sesquiterpenoids, although *Padina pavonica* (11%), *Cladostephus spongiosus* (8%), *Cystoseira sedoides* (8%), and *Dictyopteris membranacea* (6%) also have significant amounts.

**Figure 19:**
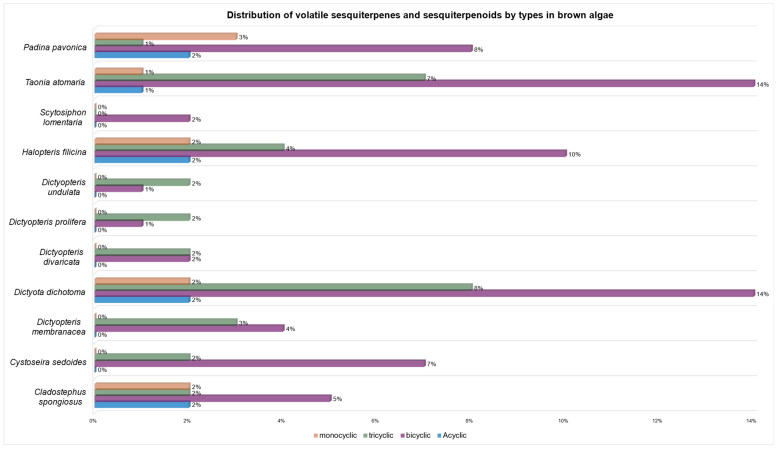
Distribution of volatile sesquiterpenes and sesquiterpenoids by number of rings in brown algae.

Among the structural classes, bicyclic and tricyclic sesquiterpenes and sesquiterpenoids were particularly abundant in *Dictyota dichotoma* (14% and 8%, respectively) and *Taonia atomaria* (14% and 7%, respectively) (Figure 19). *Padina pavonica* (8%) and *Cystoseira sedoides* (7%) are also good sources of bicyclic non-volatile sesquiterpenoids. Monocyclic and acyclic sesquiterpenes were much less frequent (from 3 to 0% in all cases), with *Padina pavonica* being the species with the highest number of monocyclic sesquiterpenes, although in a very small ratio (3%).

## 3. Conclusions

The study of sesquiterpenes in brown algae has revealed that these marine organisms are able to produce a variety of compounds with a remarkable structural diversity, mainly mono-, bi-, and tricyclic structures, such as germacranes, cadinanes, and selinanes, along with less common derivatives such as spiroaxanes and oplopanes. This structural diversity not only reflects the biosynthetic complexity of brown algae but also their ability to adapt to singular environmental conditions, suggesting a close link between algal ecology and the production of specific metabolites.

The identification of compounds with significant biological activities, such as antibacterial, antifungal, and neuroprotective properties, reinforces the potential of sesquiterpenes as a source of new therapeutic agents. In parallel, volatile sesquiterpene compounds (VOCs) play crucial roles in chemical communication, predator defense, and environmental adaptation. These VOCs show marked variability between species, month of collection, and even the extraction protocol, making the right selection of the extraction method important when seeking to obtain representative chemical profiles. Techniques such as HS-SPME, HD, MAE, and SFE significantly differ in the composition of volatile metabolites profiles, underscoring the need for standardized comparative approaches.

In summary, brown algal sesquiterpenes represent a promising field for future phytochemical and pharmacological research. The development of more advanced analytical techniques, along with the integration of molecular and genomic tools, will allow for a wider knowledge of these compounds, their biotechnological applications, and their ecological relevance. Furthermore, the study of these metabolites can provide key insights into the phylogenetic and ecological relationships within the brown algal group.

## Figures and Tables

**Table 1:** Presence of sesquiterpenes and sesquiterpenoids with monocyclic skeleton in brown algae.

Compound ^1^	*Dictyopteris* *divaricata*	*Dilophus* *fasciola*	*Padina* *gymnospora*	*Taonia atomaria*
**1**	[15]			
**2**				[16]
**3a**		[17]		
**3b**				[18]
**4**	[15]			
**5**				[18]
**6**			[19]	

^1^ See Figure 1 for structures.

**Table 2:** Presence of sesquiterpenes and sesquiterpenoids with bicyclic skeleton in brown algae.

Compound ^1^	*Dictyopteris delicatula*	*Dictyopteris divaricata*	*Dictyopteris zonaroides*	*Dilophus fasciola*	*Taonia atomaria*
**7a,7b**		[21]			
**8a,8b**		[22]			
**9**		[21]			
**10a**, **10b**		[21]			
**11**				[23]	[24]
**12**			[25]		
**13a**				[23]	
**13b**					[16]
**14**				[23]	[18]
**15**		[21]			
**16a**	[26]	[27]		[23]	[24]
**16b**		[27]			
**17a**	[26]	[27]			
**17b**					[18]
**18a**, **18b**		[28,29]			
**18c**		[29]			
**19a**, **19b**		[29]			
**20a**	[26]				
**20b**		[27]			
**20c**		[29]			
**21**		[29]			
**22**–**23**		[27]			
**24**					[30]
**25**		[30]			
**26**					[24]
**27**					[18]
**28**					[17,18]
**29**–**33**		[31]			

^1^ See Figure 2, Figure 3, Figure 4, Figure 5, Figure 6 and Figure 7 for structures.

**Table 3:** Presence of sesquiterpenoids with tricyclic skeleton in brown algae.

Compound ^1^	*Taonia atomaria*	*Taonia lacheana*
**34a**, **34b**	[16]	
**35a**, **35b**		[24]
**36**		[24]

^1^ See Figure 8 for structures.

**Table 4:** Presence of other sesquiterpenoids in brown algae.

Compound ^1^	*Dictyopteris divaricata*	*Dictyopteris zonaroides*	*Perithalia caudata*	*Dictyopteris undulata*	*Pachydictyon coriaceum*	*Sargassum polycystum*	*Padina gymnospora*
**37**					[32]		
**38**–**42**	[34]						
**43**						[35]	
**44a**	[15,33]	[36]	[37]				
**44b**		[36]					[37]
**45a**				[36,38,39,40,41,42,43,44]			
**45b**				[36,38,41,42]			
**46a**				[36,39,40,41,42]			
**46b**				[41]			
**47**				[41]			
**48**				[40,42]			
**49**				[42]			
**50**				[36,40,43]			
**51**	[34]						
**52**				[36]			
**53**	[34]			[36,38,41,42,45]			
**54**				[36,38,45]			

^1^ See Figure 9, Figure 10, Figure 11, Figure 12, Figure 13, Figure 14 and Figure 15 for structures.

**Table 5:** Volatile sesquiterpenes detected by GC/MS in brown algae (species named I–XI).

Compound	I^1^	II^2^	III^3^	IV^4^	V^5^	VI^6^	VII^7^	VIII^8^	IX^9^	X^10^	XI^11^
**Acyclic**											
*α*-farnesene (**55**)											[53]
(*E*)-*β*-farnesene (**56**)				[55]				[56]		[53]	[53]
(*E*,*E*)-farnesyl acetone (**57**)	[51]										
(*E*)-geranylacetone (**58**)				[57]							
hexahydrofarnesyl acetone (**59**)	[51]							[54]			
**Monocyclic**											
(*E*)-*α*-bisabolene (**60**)											[53]
*β*-bisabolene (**61**)											[53]
*trans*-*γ*-bisabolene (**62**)								[56]			
Ar-curcumene (**63**)								[56]			
*γ*-curcumene (**64**)				[50]							
*β*-elemene (**65**)					[58]				[59]		
*γ*-elemene (**66**)										[53]	
germacrene-4-ol (**67**)	[51]										
germacra-4(15),5,10(14)-trien-1α-ol (**68**)		[52]									
germacrene A (**1**)				[55]							
germacrene B (**69**)								[56]			
germacrene C (**70**)				[50]							
germacrene D (**2**)	[51]	[52]	[49]	[50,56,57]	[58]			[54,56]		[53]	
*α*-humulene (**71**)				[50]							[53]
**Bicylic**											
bicyclogermacrene (**72**)				[56]				[56]		[53]	
9-*epi*-(*E*)-caryphyllene (**73**)				[55]							
gleenol (**28**)	[51]	[52]	[49]	[50,55]				[54]		[53]	
(*E*)-*β*-guaiene (**74**)											[53]
*γ*-gurjunene (**75**)		[52]		[55]						[53]	
pachydictyol A (**37**)											[53]
isopachydictyol A (**76**)								[54]			[53]
*β*-santalene (**77**)											[53]
*epi-β*-santalene (**78**)											[53]
*trans-α*-bergamotene (**79**)										[53]	[53]
junenol (**80**)										[53]	[53]
*α*-selinene (**81**)				[57]							
*δ*-selinene (**82**)								[56]			
α-amorphene (**83**)			[49]	[50,55]				[56]		[53]	
*γ*-amorphene (**84**)				[55]						[53]	
cadalene (**15**)		[52]									
*epi*-bicyclosesquiphellandrene (**85**)	[51]		[49]	[50]						[53]	[53]
cadina-3,5-diene (**86**)										[53]	[53]
cadina-4,9-diene (**87**)	[51]										
cadina-1,4-diene (**88**)			[49]	[50]							
*trans*-cadina-1,4-diene (**89**)		[52]		[50,55]				[56]		[53]	
4,10(14)-cadinadien-8*β*-ol (**90**)										[53]	[53]
α-cadinene (**91**)								[56]		[53]	
*β*-cadinene (**92**)				[50,55,56,57]				[56]		[53]	
*γ*-cadinene (**93**)	[51]			[50,55,57]				[56]		[53]	
*δ*-cadinene (**11**)	[51]		[49]	[50,55,56,57]	[58]	[58]	[58]	[54,56]	[59]	[53]	
α-cadinol (**94**)	[51]	[52]						[56]		[53]	
*δ*-cadinol (**95**)	[51]									[53]	
*τ*-cadinol (**96**)	[51]			[50]						[53]	
α-calacorene (**97**)		[52]	[49]	[50]				[56]		[53]	
cis-calamenene (**98**)		[52]									[53]
*trans*-calamenene (**99**)											[53]
cubenol (**16a**)		[52]		[50,55]	[58]			[54]			
*epi*-cubenol (**100**)				[50,55]					[59]		
*cis*-muurola-3,5-diene (**101**)				[57]							
*trans*-muurola-3,5-diene (**102**)				[55]							
*cis*-muurola-4(15),5-diene (**103**)				[55]						[53]	[53]
muurola-4,9-diene (**104**)				[57]							
*α*-muurolene (**105**)				[55,56,57]				[54,56]		[53]	
*γ*-muurolene (**106**)				[50,55,57]				[56]		[53]	
α-muurolol (**107**)				[55]						[53]	
*τ*-muurolol (**108**)								[56]			
zonarene (**12**)	[51]		[49]	[55]				[54]		[53]	
*epi*-zonarene (**109**)		[52]						[56]		[53]	
14-nor-cadin-5-en-4-one (**110**)		[52]									
*α*-eudesmol (**111**)		[52]									
*β*-oplopenone (**112**)		[52]									
**Tricyclic**											
alloaromadendrene (**113**)										[53]	[53]
aromandrene (**114**)								[56]		[53]	
*β*-gurjunene (**115**)				[50,57]							
*α*-cedrene (**116**)				[57]							
*β*-cedrene (**117**)				[57]							
*β*-patchoulene (**118**)										[53]	
*β*-bourbonene (**119**)	[51]	[52]	[49]	[50,55,56,57]				[54,56]		[53]	
cubebol (**34**)				[55]						[53]	
1,10-di-*epi*-cubebol (**120**)			[49]							[53]	
*α*-copaene (**121**)			[49]	[50,55,56,57]	[58]	[58]	[58]	[56]		[53]	
*β*-copaene (**122**)				[55]						[53]	
*α*-cubebene (**123**)		[52]	[49]	[50,55,57]	[58]	[58]	[58]	[54,56]		[53]	
*β*-cubebene (**124**)	[51]	[52]	[49]	[50,55]				[54,56]		[53]	
cyclosativene (**125**)				[55]						[53]	
cycloisosativene (**126**)								[56]			
*cis*-4,10-epoxy-amorphane (**127**)				[55]							
sativene (**128**)				[55]							
*α*-ylangene (**129**)								[56]		[53]	
*β*-ylangene (**130**)				[55]						[53]	[53]

I^1^—Cladostephus spongiosus; II^2^—Cystoseira sedoides; III^3^—Dictyopteris membranacea; IV^4^—Dictyota dichotoma; V^5^—Dictyopteris divaricata; VI^6^—Dictyopteris prolifera; VII^7^—Dictyopteris undulata; VIII^8^—Halopteris filicina; IX^9^—Scytosiphon lomentaria; X^10^—Taonia atomaria; XI^11^—Padina pavonica.

## Data Availability

No new data were created or analyzed in this study. Data sharing is not applicable to this article.

## Supplementary Materials

The following supporting information can be downloaded at: https://www.mdpi.com/article/10.3390/md23050210/s1, Figure S1. Acyclic volatile sesquiterpenes and sesquiterpenoids; Figure S2. Monocyclic volatile sesquiterpenes and sesquiterpenoids; Figure S3. Bicyclic volatile sesquiterpenes and sesquiterpenoids; Figure S4. Tricyclic volatile sesquiterpenes and sesquiterpenoids.
