# Supplementary material for: Sesquiterpenes from Brown Algae
Source: Mar Drugs. 2025 May 15;23(5):210. doi: 10.3390/md23050210 (PMC12113178; doi:10.3390/md23050210)
Supplement: Supplementary file 1 [file marinedrugs-23-00210-s001.zip › marinedrugs-3616063-supplementary.pdf]

Supplementary Material

Sesquiterpenes from Brown Algae

Irene Moreno-Gutiérrez, Sonia Berenguel-Gómez, Manuel Muñoz-Dorado, Míriam Álvarez-Corral * and Ignacio Rodríguez-García *

[1] Organic Chemistry, University of Almería, CIAIMBITAL. E04120 Almería, Spain .); img823@ual.es (I.M. -G.); sbg479@ual.es (S.B.G); mdorado@ual.es (M.M.-D.); malvarez@ual.es (M.A.-C.); irodrigu@ual.es (I.R.-G.)

* Correspondence: irodrigu@ual.es

VOLATILE SESQUITERPENES

Figure S1. Acyclic volatile sesquiterpenes and sesquiterpenoids 2

Figure S2. Monocyclic volatile sesquiterpenes and sesquiterpenoids............................... 2

Figure S3. Bicyclic volatile sesquiterpenes and sesquiterpenoids 3

Figure S4. Tricyclic volatile sesquiterpenes and sesquiterpenoids 5

Figure S1. Acyclic volatile sesquiterpenes and sesquiterpenoids

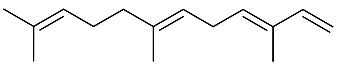

55
α-farnesene

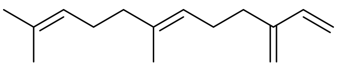

56
(*E*)-β-farnesene

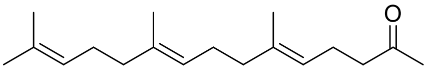

57
(*E,E*)-farnesyl acetone

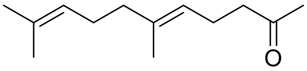

58
(*E*)-geranylacetone

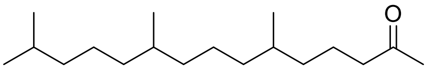

59
hexahydrofarnesyl acetone

Figure S2. Monocyclic volatile sesquiterpenes and sesquiterpenoids

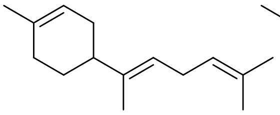

60
(*E*)-α-bisabolene

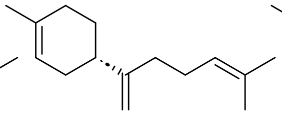

61
β-bisabolene

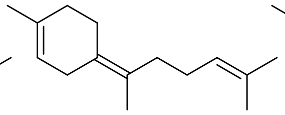

62
trans-γ-bisabolene

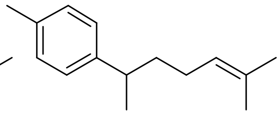

63
ar-curcumene

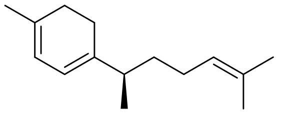

64
γ-curcumene

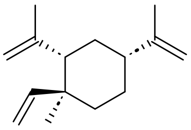

65
β-elemene

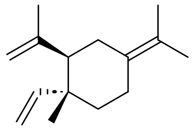

66
γ-elemene

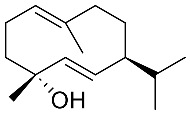

67
germacrene-4-ol

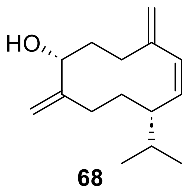

68
germacra-4(15),5,10(14)-trien-1α-ol

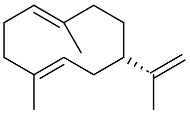

1
germacrene A

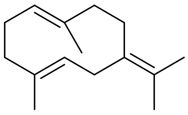

69
germacrene B

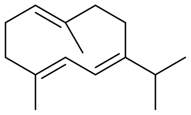

70
germacrene C

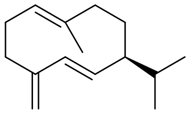

2
germacrene D

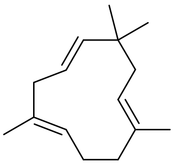

71
α-humulene

Figure S3. Bicyclic volatile sesquiterpenes and sesquiterpenoids

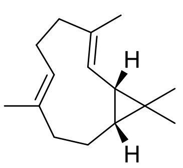

72
bicyclogermacrene

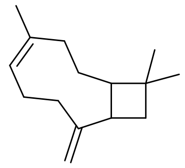

73
9-epi-(*E*)-caryphyllene

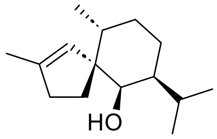

28
gleenol

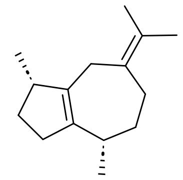

74
(*E*)-β-guaiene

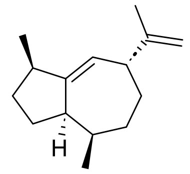

75
γ-gurjunene

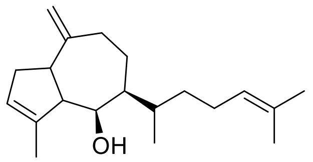

37
pachydictyol A

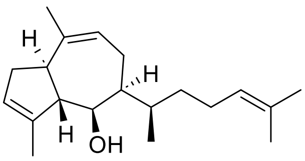

76
isopachydictyol A

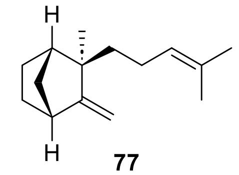

77
β-santalene

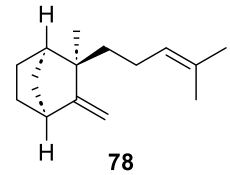

78
epi-β-Santalene

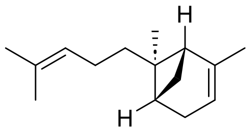

79
trans-α-bergamotene

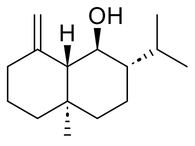

80
junenol

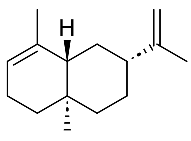

81
α-selinene

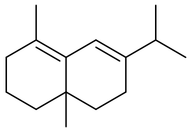

82
δ -selinene

Figure S3. Bicyclic volatile sesquiterpenes and sesquiterpenoids (Cont.)

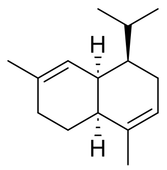

83
α-amorphene

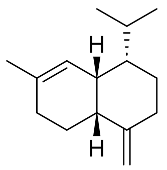

84
γ-amorphene

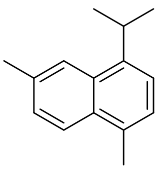

15
cadalene

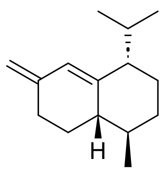

85
epi-bicyclosesquiphellandrene

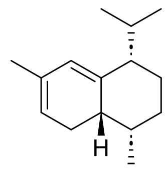

86
cadina-3,5-diene

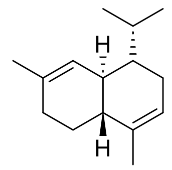

87
cadina-4,9-diene

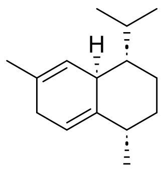

88
cadina-1,4-diene

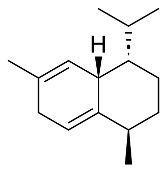

89
trans-cadina-1,4-diene

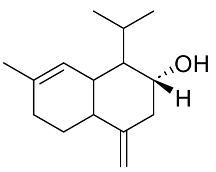

90
4,10(14)-cadinadien-8β-ol

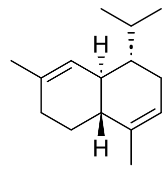

91
α-cadinene

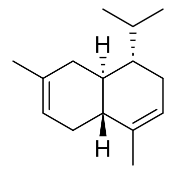

92
β-cadinene

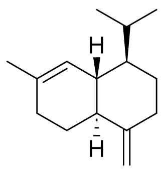

93
γ-cadinene

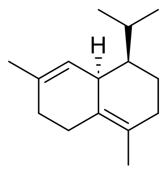

11
δ-cadinene

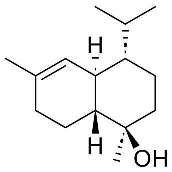

94
α-cadinol

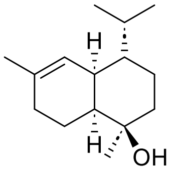

95
δ-cadinol

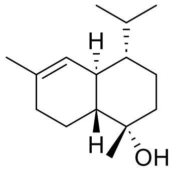

96
τ-cadinol

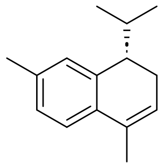

97
α-calacorene

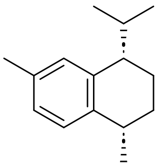

98
cis-calamenene

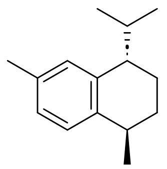

99
trans-calamenene

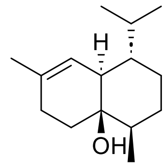

16a
cubenol

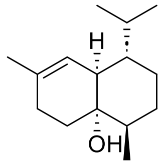

100
epicubenol

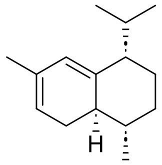

101
cis-muurola-3,5-diene

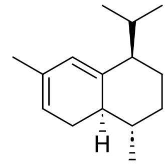

102
trans-muurola-3,5-diene

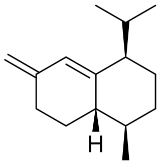

103
cis-muurola-4(14),5-diene

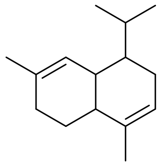

104
muurola-4,9-diene

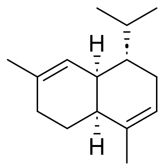

105
α-muurolene

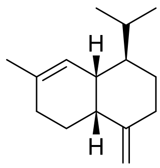

106
γ-muurolene

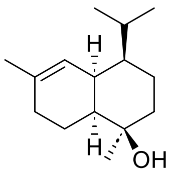

107
α -muurolol

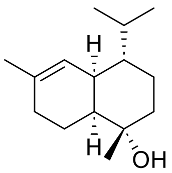

108
τ-muurolol

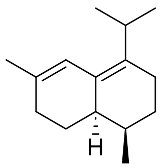

12
zonarene

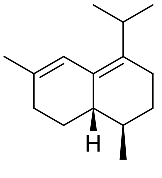

109
epi-zonarene

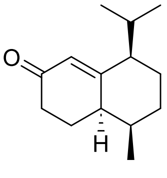

110
14-nor-cadin-5-en-4-one

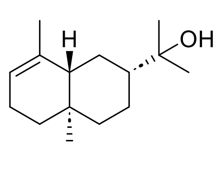

111
α-eudesmol

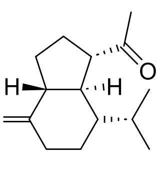

112
β-oplopenone

Figure S4. Tricyclic volatile sesquiterpenes and sesquiterpenoids

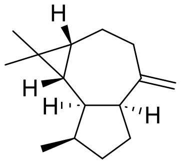

113
alloaromadendrene

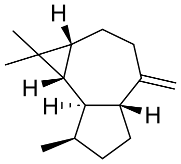

114
aromadendrene

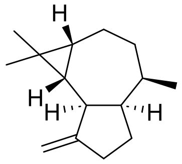

115
β-gurjunene

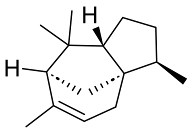

116
α-cedrene

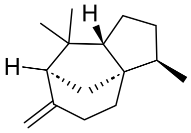

117
β-cedrene

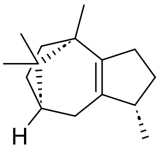

118
β-patchoulene

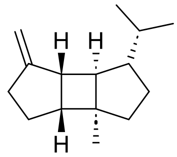

119
β-bourbonene

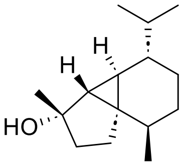

34
cubebol

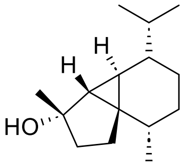

120
1,10-di-*epi*-cubebol

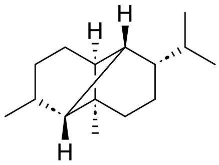

121
α-copaene

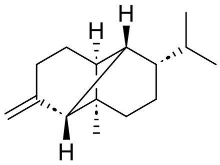

122
β-copaene

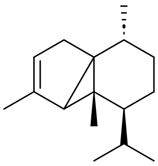

123
α-cubebene

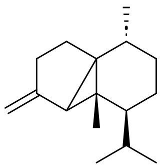

124
β-cubebene

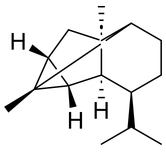

125
cyclosativene

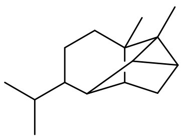

126
cycloisosativene

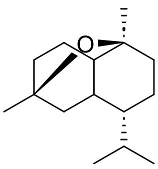

127
cis-4,10-epoxy-Amorphane

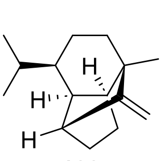

128
sativene

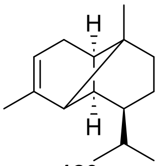

129
α-ylangene

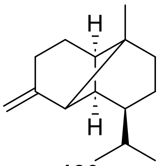

130
β-ylangene
